# Appropriateness of carpal tunnel syndrome management compared with the AAOS appropriate use criteria: A retrospective review across various specialties

**DOI:** 10.1016/j.amsu.2022.104140

**Published:** 2022-07-12

**Authors:** Loay A. Salman, Rand Y. Omari, Isam S. Moghamis, Ashraf T. Hantouly, Ghalib Ahmed

**Affiliations:** a Orthopedic Surgery Department, Hamad General Hospital, Hamad Medical Corporation, PO Box 3050, Doha, Qatar; b Plastic and Reconstructive Surgery Department, Hamad General Hospital, Hamad Medical Corporation, PO Box 3050, Doha, Qatar; c Nuffield Department of Orthopaedics, Rheumatology and Musculoskeletal Sciences, Botnar Research Centre, University of Oxford, Windmill Road, Oxford, OX3 7LD, UK

**Keywords:** Carpal tunnel syndrome, Hand surgery: AAOS, AUC, Appropriateness, Management

## Abstract

**Purpose:**

Carpal tunnel syndrome (CTS) is a common peripheral nerve entrapment disorder among adults that causes upper-extremity disability. The American Academy of Orthopedic Surgeons (AAOS) developed an evidence-based appropriate use criteria (AUC) for the management of CTS. This study aimed to assess the appropriateness of our practice and the usability of the AUC through comparing the actual management provided at our institution with that recommended by the AUC.

**Methods:**

A retrospective review of the electronic medical records at our hospital was performed between 1 Jan 2016 and 31 Dec 2019. Data were collected by two authors independently. The collected data were input into the AUC application to determine the rate of the appropriateness of the treatments. Afterwards, the agreement between the actual treatment provided and the AUC recommendation was assessed. The primary outcome was the appropriateness rate. Descriptive statistics such as the mean, range and percentage were used to summarize the patients’ demographics and treatment options.

**Results:**

There were 127 patients (169 interventions), with a mean age of 50.18 years (range, 24–85 years). Most of the included patients were females, 78% (99). Obesity was the most frequent risk factor 64.5% (82), and bilateral wrist involvement was the most common presentation 58% (74). The overall appropriateness rate and agreement with the AUC recommendations among all interventions was 84%. A sub-analysis of carpal tunnel surgical release across different surgical specialities showed appropriateness rates of 88%, 89%, 54% in orthopaedic surgery, neurosurgery and plastic surgery teams, respectively (Chi^2^ 19.54, P-Value 0.000613).

**Conclusion:**

This study demonstrated that most of the treatments provided at our institution were appropriate and in agreement with the AUC recommendations. Additionally, the AUC for carpal tunnel syndrome is a valuable and practical tool that can be applied in clinical settings.

**Level of evidence:**

Retrospective study, level IV.

## Highlights

• The first study to assess the AAOS Appropriate use criteria for the management of carpal tunnel syndrome.
• The usability of the AUC in carpal tunnel syndrome was evaluated against a cohort of patients in a single center.
• A significant difference was observed in the appropriateness rate across orthopaedics, neurosurgery, and plastics surgery.
• The AUC of CTS management is a valuable and practical tool that can be applied in clinical settings.

## 1 Introduction

Carpal tunnel syndrome (CTS) is a common peripheral nerve entrapment disorder among adults that causes upper-extremity disability. The estimated prevalence of CTS in the general population is 1–5% [1,2]. The diagnosis is mainly clinical for patients with characteristic signs and symptoms, classically, pain or paresthesia confined to the median nerve distribution [3]. Electrodiagnostic testing can aid in confirming or excluding the diagnosis, in addition to identifying the severity of nerve compression which could necessitate surgical intervention [4]. Appropriate management options include nonoperative measures with splinting and corticosteroid injections, or surgical intervention, depending on the severity of CTS [5].

In 2016, the American Academy of Orthopedic Surgeons (AAOS) developed an evidence-based clinical practice guideline for the management of CTS to assist clinicians in managing this condition based on the best evidence available [6]. Following these guidelines, the AAOS approved the appropriate use criteria (AUC) for the management of CTS. The AUC generates treatment recommendations based on a hierarchy of several diagnostic criteria to aid in the decision-making process. An appropriateness rating is yielded for each of the following six interventions: Carpal tunnel release (operative treatment), splinting (non-operative treatment), steroid injection (non-operative treatment), Oral steroids or ketoprofen phonophoresis (non-operative treatment), investigate further (electrodiagnostic study), or investigate alternative diagnosis [7].

To the best of our knowledge, no previous studies have investigated the value of the AUC as a tool for the management of CTS in clinical practice. This study aimed to assess the appropriateness and usability of the AUC by comparing the actual management option provided at our institution with that recommended by the AUC. We hypothesize that our practice aligns with the AUC recommendations of carpal tunnel syndrome management and is associated with an excellent appropriateness rate.

## 2 Methodology

The Institutional Medical Research Centre approved this cohort retrospective study (X), and it was conducted at a single tertiary academic care center, which is accredited by Joint Commission International (JCI) and Accreditation Council of Graduate Medical Education-International (ACGME-I). This study was reported in accordance with the STROCSS guidelines [8] and was registered in ClinicalTrials.gov with the unique identifying number X.

## 3 Eligibility criteria

**Graph 1:**
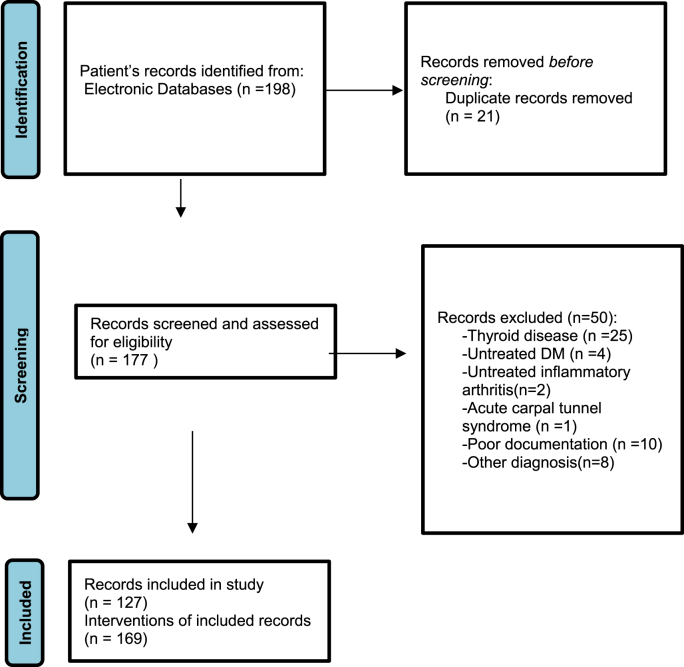
Inclusion & Exclusion criteria.

The eligibility criteria were according to the AUC criteria for CTS management in adults. All adult patients (≥18 years) who were diagnosed with CTS between 2016 and 2019 were included. The exclusion criteria were acute carpal tunnel syndrome, untreated inflammatory arthritis, untreated diabetes, thyroid disease, pernicious anemia, patients with a known space-occupying lesion in the carpal tunnel and patients who failed treatment after surgery. Graph 1 shows the complete inclusion and exclusion process.

**Fig. 1:**
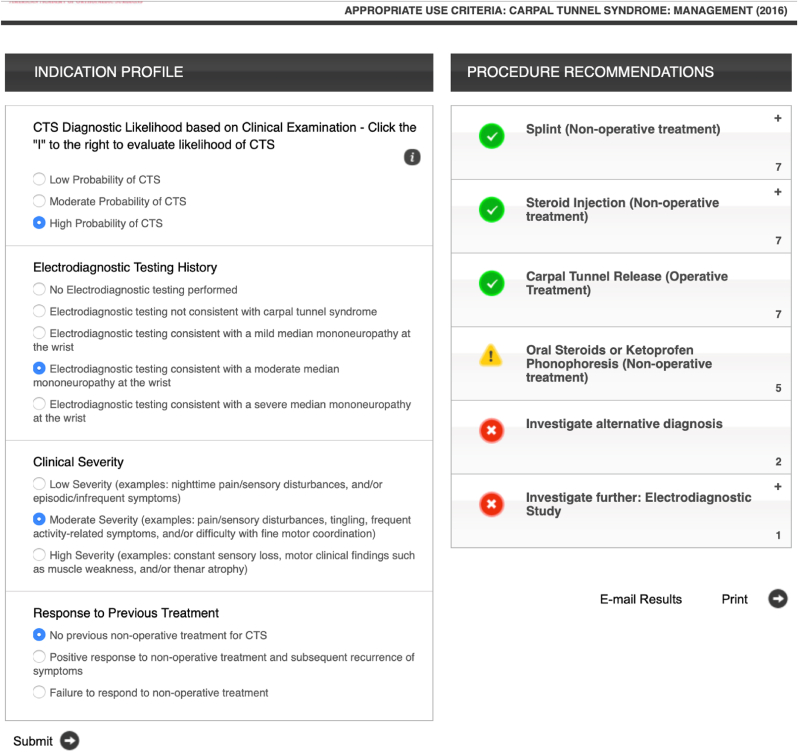
Data entry and interpretation on the AAOS appropriate use criteria free web application.

## 4 Data source and collection

The data source was our institution's electronic medical records. The database was searched from 1 Jan 2016 until 31 Dec 2019 with the keywords “Carpal tunnel syndrome” and “median nerve palsy” and their derivatives to identify all potential cases. Two authors performed the search and data collection independently. The baseline variables that were collected included age, gender, comorbidities, occupation, hand dominance, specialty of operating surgeon, follow-up duration, AUC generated recommendation and the four AUC variables including; Likelihood, clinical severity, EMG testing and previous treatment. We also collected any postoperative symptoms persistence, recurrence of symptoms, joint stiffness, and infection.

The AUC application for carpal tunnel syndrome management requires four patients’ variables to generate appropriateness ratings for six different treatment options. Each procedure is rated as appropriate, maybe appropriate, or rarely appropriate. The six procedures include splint, steroid injection, carpal tunnel release, oral steroids or Ketoprofen phonophoresis, investigation of alternative diagnoses, and further investigation using an electro-diagnostic study. Fig. 1 shows the interface of the AAOS appropriate use criteria free web application.

To judge the usability of the AUC for the management of carpal tunnel syndrome, the required parameters of each patient were collected and entered into the AUC application by two independent authors to generate the appropriateness rating of the provided treatment for each patient. Afterwards, the agreement between the AUC recommendations and the actual treatment that was provided at our institution was assessed.

## 5 Statistical analysis

The statistical software (IBM SPSS version 24; SPSS Inc., Chicago, IL, USA) was used for data analysis. Measures of central tendency such as the mean, range and percentage were used to summarize the patients’ demographics and management options. In addition, chi^2^ test was utilized for categorical data, and a p-value < 0.05 was considered significant.

The appropriateness rating (appropriate, maybe, or rarely appropriate) for each treatment was described with percentages. Similarly, the agreement of the treatments implemented at our institution with the AUC recommendations was expressed as a proportion.

A Pearson's correlation coefficient was calculated to assess and compare the validity of the data collection process performed by two authors, and an intraclass correlation coefficient (ICC) > 0.75 was considered to indicate excellent agreement.

No power analysis was performed because all patients who met the inclusion criteria were included in this study. Furthermore, a subgroup analysis within the same cohort compared the appropriateness rates of surgical release of patients across different surgical specialties using chi^2^ test.

## 6 Results

A total of 127 patients who met our inclusion criteria with an average follow-up of 3.19 months were included in the study. The mean age was 50.18 years; Patients were primarily females, 78% (99), and 22% (28) males. Obesity was the most common risk factor 64.5% (82), followed by diabetes 38.6% (49). More than half of the cases were managed by orthopedic surgeons 54%, followed by plastic surgeons 32% and neurosurgeons in only 14%. Also, 74 patients (58%) had bilateral CTS involvement. The average CTS-6 was 13.16, and 64% of the patients had moderate disease severity. 82% (104) of patients had no prior treatment of the condition, while 14% (18) failed previous treatment and the remaining 4% (5) had a positive response to previous treatment. (Table 1).

**Table 1:** Patients’ demographics.

Patient's characteristics	Frequency	Percentage
Age (years)		
Mean	50.18	
Range	24–85	
**Gender**		
Male	28	22%
Female	99	78%
**Surgeon's specialty**		
Orthopaedics	68	54%
Neurosurgery	18	14%
Plastic surgery	41	32%
**Avg Follow-up (months)**	3.19	
**Wrist affected**		
Right	30	24%
Left	23	18%
Bilateral	74	58%
**Risk factors**		
Obesity	82	64.5%
DM	49	38.6%
High hand/wrist repetition rate	19	15%
Pregnancy	8	6%
Alcohol	4	3.2%
Smoking	10	8%
None	18	14%
**CTS-6 Avg**	13.16	
**Electrodiagnostic testing**		
Not performed	8	6%
Mild	12	9.5%
Moderate	58	46%
Severe	49	38.5%
**Clinical severity**		
Low	39	31%
Moderate	82	64%
High	6	5%
**Response to previous treatment**		
No previous treatment	104	82%
Failure to respond	18	14%
Positive response	5	4%

Compared to the AAOS-published AUC, our overall management was “appropriate” in 84% of the cases, “maybe appropriate” in 21%, and “rarely appropriate” in 4%. CT surgical release was appropriate for 78% of the cases, while splinting and steroid injections were appropriate in all the patients (100%) who underwent these treatment modalities. Furthermore, no patients were further investigated for CTS or alternative diagnoses as per the AUC. (Table 2).

**Table 2:** AUC treatment options, rate of appropriateness, and rate of agreement (main table all interventions).

	Number of interventions	Appropriate	Maybe	Rarely
Overall	169	141 (84%)	21 (12%)	7 (4%)
CT release	127	99 (78%)	21 (16.5%)	7 (5.5%)
Splint	23	23 (100%)	0	0
Steroid Injection	19	19 (100%)	0	0

Interestingly, a sub-analysis of carpal tunnel surgical release across different surgical specialties showed appropriateness rates of 89%, 88%, 54% in neurosurgery, Orthopedics and plastics surgery teams, respectively (Chi^2^ 19.54, P-Value 0.000613) (Table 3).

**Table 3:** Appropriateness of carpal tunnel surgical release across different surgical specialties.

Speciality	Overall	Appropriate	Maybe	Rarely
Orthopedics	68	60 (88%)	6 (9%)	2 (3%)
Neurosurgery	18	16 (89%)	1 (5.5%)	1 (5.5%)
Plastics	41	22 (54%)	15 (36%)	4 (10%)
				
**Chi**^**2**^	19.5482	**P-Value**	0.000613	

## 7 Discussion

This study evaluated the appropriateness of CTS management at a tertiary care hospital over four years. The most essential ascertainment of our study was that the use of the AUC application made selecting an appropriate management for each patient relatively simple and straightforward. Previous studies have assessed the reliability of AUC as a clinical tool in various conditions such as knee osteoarthritis and hip fractures management [[9], [10], [11]], yet no one has investigated the AUC in carpal tunnel syndrome before.

In our patients, most AUC-recommended management options were performed with a predominance of CT release, although none of the orthopedic surgeons, neurosurgeons, or plastic surgeons at our institute had used the AUC preoperatively. This finding demonstrated the consensus regarding management of CTS at our institute with evidence-based indications.

The provided conservative management options for CTS at our institution were appropriate and in agreement with the AUC recommendations in all cases including splinting and steroid injection. In the literature, corticosteroid injection has shown improvement in 32% of affected patients and precluded the need for surgery. Corticosteroid injection should therefore be considered prior to surgical intervention. Furthermore, non-surgical splinting by an occupational therapist has also been shown to improve symptoms of carpal tunnel syndrome [12,13]. Of the 127 patients included in our study, only 42 patients were managed conservatively with 100% appropriateness. This is perhaps due to overestimation of the importance of surgical intervention by some surgeons.

The appropriateness of carpal tunnel surgical release varied across different surgical specialties with 89%, 88%, and 54% in neurosurgery, orthopedic surgery, and plastic surgery, respectively. This might be explained by the inconsistent sample size across different specialties. In a systematic review done by Shi, Q. et al., they assessed seven studies in their review including five RCTs and two controlled trials to investigate whether surgical management of CTS was more beneficial than non-surgical management. They found that surgical treatment had a superior benefit, in symptoms and function, at six and twelve months postoperatively compared to non-surgical management at these intervals [14]. However, conservative interventions are beneficial, and effects usually plateau within three months, therefore the traditional approach to use a trial of conservative management in patients with mild and moderate or transient CTS is supported by evidence [15,16].

In the AUC, one of the key components of deciding CTS management recommendation is determining the likelihood of CTS which is done through CTS-6. CTS-6 is a validated 26-point weighted scale that takes into consideration two symptoms and four signs during a patient encounter to determine probability of CTS. These criteria improve the consistency of the diagnosis of CTS leading to more effective treatment [17].

Electrodiagnostic studies remain an integral part of CTS management, a tool appreciated by the AUC. The majority of our patients (94%) underwent electrodiagnostic studies prior to commencing management. Which is considered as a valid and reliable means of confirming the clinical diagnosis of CTS as well as quantitative measure of the physiological function of the median nerve. This in turn may be used to guide management and determine prognosis [18,19].

While it is not part of the AAOS AUC recommendations, ultrasound (US) as a CTS diagnostic and therapeutic modality has gained immense recognition. Recent literature shows that ultrasound can be as effective as other modalities in establishing a diagnosis of CTS, particularly with anatomical variations [20]. Also, Kamel SI et al. showed that a minimally invasive ultrasound-guided carpal tunnel release was as safe and effective as traditional surgery, with significant improvements in long term functional outcomes [21,22].

One of the major strengths of this study was promoting evidence-based medicine by assessing the applicability of AUC as a tool in clinical practice. To the best of our knowledge, this study was the first to clinically compare CTS management against the current standard of care and recommendations produced by AAOS AUC. The findings of this work can be reproduced and implemented in other institutes to enhance evidence-based practice with the best treatment outcomes in CTS patients.

Owing to the retrospective design of our study, several limitations must be acknowledged. This includes the inevitable selection bias and the absence of randomized comparative groups. However, a sub-analysis based on the surgeon's speciality within the same cohort was meant to reduce this effect. Also, one of the drawbacks of implementing AAOS AUC as a standard tool is its lack of some interventional modalities recently described and used in the literature, such as ultrasound imaging. Furthermore, a longer follow-up period would have enabled us to observe the long-term outcomes and potential complications; however, this did not affect the main objective of this study which is to assess our management choices compared to the AUC and check its usability.

## 8 Conclusion

This study demonstrated that most of the treatments provided at our institution were appropriate and in agreement with the AUC recommendations. Additionally, the AUC for carpal tunnel syndrome is considered an easy and practical tool that can be applied in clinical settings to guide the management of carpal tunnel syndrome.

## Ethical approval

The Institutional Medical Research Centre at Hamad medical corporation approved this cohort retrospective study (MRC-01-22-085)

## Sources of funding

None.

## Author contribution

LS was involved with data collection, analysis, drafting, reviewing, and editing of the manuscript. RO, IM, AH were involved with data collection, manuscript writing and editing. GA is the senior author who was involved with reviewing and editing of the final manuscript and overall supervision of the study. All contributors met the ICMJE and journal criteria for authorship and approved the final manuscript.

## Research registration unique identifying number (UIN)

Name of the registry: ClinicalTrials.gov.

Unique Identifying number or registration ID: NCT05275816.

Hyperlink to your specific registration (must be publicly accessible and will be checked):

Appropriateness of Carpal Tunnel Syndrome Management Compared With the AAOS Appropriate Use Criteria.

## Guarantor

1)Loay A. Salman.

## Provenance and peer review

Not commissioned, externally peer-reviewed.

## Declaration of competing interest

None.
